# Utilisation of Carp Skin Post-Production Waste in Binary Films Based on Furcellaran and Chitosan to Obtain Packaging Materials for Storing Blueberries

**DOI:** 10.3390/ma14247848

**Published:** 2021-12-18

**Authors:** Magdalena Janik, Ewelina Jamróz, Joanna Tkaczewska, Lesław Juszczak, Piotr Kulawik, Michał Szuwarzyński, Karen Khachatryan, Pavel Kopel

**Affiliations:** 1 Department of Chemistry, Faculty of Food Technology, University of Agriculture, ul. Balicka 122, 30-149 Kraków, Poland; janikmagda090@gmail.com (M.J.); ewelina.jamroz@urk.edu.pl (E.J.); karen.khachatryan@urk.edu.pl (K.K.); 2 Department of Animal Product Technology, Faculty of Food Technology, University of Agriculture, ul. Balicka 122, 30-149 Kraków, Poland; tkaczewska@gmail.com (J.T.); kulawik.piotr@gmail.com (P.K.); 3 Department of Food Analysis and Evaluation of Food Quality, University of Agriculture, ul. Balicka 122, 30-149 Krakow, Poland; rrjuszcz@cyf-kr.edu.pl; 4 Academic Centre for Materials and Nanotechnology, AGH University of Science and Technology, Al. Mickiewicza 30, 30-059 Kraków, Poland; szuwarzy@agh.edu.pl; 5 Department of Inorganic Chemistry, Faculty of Science, Palacky University, 17. listopadu 12, 77146 Olomouc, Czech Republic

**Keywords:** biopolymer films, gelatin hydrolysate, furcellaran, chitosan

## Abstract

The aim of the study was to develop and characterise an innovative three-component biopolymer film based on chitosan (CHIT), furcellaran (FUR) and a gelatin hydrolysate from carp skins (*Cyprinus carpio*) (HGEL). The structure and morphology were characterised using the Fourier transform infrared spectroscopy (FT-IR) and atomic force microscopy (AFM). The FT-IR test showed no changes in the matrix after the addition of HGEL, which indicates that the film components were compatible. Based on the obtained AFM results, it was found that the addition of HGEL caused the formation of grooves and cracks on the surface of the film (reduction by ~21%). The addition of HGEL improved the antioxidant activity of the film (improvement by up to 2.318% and 444% of DPPH and FRAP power, respectively). Due to their properties, the tested films were used as active materials in the preservation of American blueberries. In the active films, the blueberries lost mass quickly compared to the synthetic film and were characterised by higher phenol content. The results obtained in this study create the opportunity to use the designed CHIT–FUR films in developing biodegradable packaging materials for food protection, but it is necessary to test their effectiveness on other food products.

## 1. Introduction

In recent years, there has been a rapid increase in the world’s population and fast economic development related to this increase, which further leads to deepening of many global problems. These particularly concern environmental pollution, consumption of non-renewable energy sources and emission of harmful compounds. Currently, the majority of packaging materials used, mainly for food products, is made of plastics from non-renewable fossil sources. This leads to an increased degree of pollution within the natural environment with plastics, the emission of harmful compounds emitted during the disposal of plastics and a reduction in natural resources [1]. Moreover, enormous amounts of food are currently being wasted worldwide. According to FAO data, approximately 1.3 billion tonnes of the food produced is wasted annually. The solution to this critical situation may be biopolymer materials. The development of innovative packaging materials based on biopolymers has been carried out for many years and it is still an area of interest for many scientists. The use of natural polysaccharides to develop modern packaging may be a revolutionary alternative to petroleum-based packaging materials made of non-recyclable and non-biodegradable plastics [2]. The coatings obtained from some polysaccharides, thanks to their linear structure, are tough, flexible and show good film-forming properties [3]. Despite all the advantages of using polysaccharides, their properties are different than those of packaging materials made from plastic. Some disadvantages, mainly related to their low water resistance, reduced barrier and mechanical properties, hinder their wider application and commercialisation [4,5]. Considering that single-component films often do not meet the requirements for application in packaging of food products, research is currently being conducted around the world to improve the properties of films by combining them with other polymers, using various types of additives, nanocomposites and cross-linking agents [6]. The great need to improve single-component films generates new ideas and projects to improve their properties, necessary for application and commercialisation as new food packaging materials. Complexing biopolymers is considered to be a promising route for the production of materials with “tailored” properties in the industry [7]. In this article, attention is paid to two polysaccharides with interesting properties. Furcellaran (FUR) is a natural polysaccharide obtained from the extract of red seaweed (*Furcellaria lumbricalis*). It exhibits properties such as non-toxicity, biodegradability, biocompatibility, water-solubility, and exceptional gel-forming ability. For this reason, it is gaining increased popularity as a biopolymer used in the development of films and coatings applied as packaging materials for food products [8]. Furcellaran can be used as a food additive [9]. Currently, many complexes based on furcellaran have been developed and tested, including furcellaran-based films with polysaccharides and proteins, with the addition of essential oils and plant extracts [10]. In turn, chitosan (CHIT) is a polysaccharide obtained by alkaline deacetylation of chitin, commonly found in the exoskeleton of insects and crustaceans [11]. Due to its biological and physical properties, it is an interesting film-forming base. Due to its unique molecular structure, it is insoluble in water but soluble in dilute acids [12]. Chitosan is one of the most abundant biopolymers following cellulose and starch. Chitosan-based materials are widely used in various fields because of their biological and physical properties in terms of biocompatibility, biodegradability, antimicrobial ability, and ease of film formation. In addition, for this polysaccharide, different film variants have been developed, tested and implemented in the area of food packaging [13]. Chitosan-based films have been studied for years due to their possible use in the food industry as food packaging materials in the form of pure chitosan films, chitosan films combined with other biopolymers or synthetic polymers [14] and films containing nanoparticles, e.g., zinc oxide (ZnO) [15] or plant extracts [16].

An equally important issue is the oxidation of food during its storage. Oxidative processes occurring in food products are a key problem in food technology, as they cause deterioration of products’ sensory quality, reducing their nutritional value [17]. Substances with antioxidant properties can be obtained from food industry waste. For example, fish skin gelatin can be further hydrolysed to produce gelatin hydrolysates with different biological properties, including antioxidant, which make them ideal biologically-active component of edible films [18]. The inclusion of antioxidants in polymer films can significantly extend the shelf-life of products packed in them. The gelatin hydrolysate used in this study has antioxidant properties, which have been demonstrated in the research by Jancikova, et al. [19], in which a film was created on the basis of furcellaran and gelatin with the addition of a gelatin hydrolysate to obtain edible films with antioxidant properties.

In an earlier work, the FUR/CHIT matrix was developed and characterised [20]. This research was focused on imparting active properties onto the innovative binary film based on chitosan (CHIT) and furcellaran (FUR) by enriching it with active ingredient—gelatin hydrolysate (HGEL) obtained from carp skins. To the best of our knowledge, there is no existing literature data describing the production and characteristics of three-component films based on chitosan and furcellaran with the addition of a protein component. The second objective of this study was to check the properties of the newly-formed active complexes through in vitro and in vivo analyses on a food product. The obtained films were used as active packaging materials in the storage of American blueberries.

## 2. Materials and Methods

### 2.1. Materials

Furcellaran (Est-Agar, Karla Village, Estonia) with a molecular weight of 295.1 kDa and chitosan with a molecular weight of ~890 kDa, and deacetylation degree of ≥90% (POL-AURA, Zabrze, Poland), were used as materials for coating preparation. No further purification was performed on any of the used chemical reagents. In the case of fruit preservation experiments, blueberries (*Vaccinium corymbosum*) were obtained from a local store (Kraków, Poland). HGEL was prepared according to the method described by Tkaczewska, et al. [21], without modifications.

### 2.2. Methods

#### 2.2.1. Preparation of Furcellaran-Chitosan Films with the Addition of Gelatin Hydrolysate

The procedure for obtaining the FUR/CHIT film in the ratio of 1:9 has been presented in an earlier study [20]. In brief, both solutions of FUR (2% aqueous solution) and CHIT (2% in 2% acetic acid solution) were prepared individually. The CHIT solution required stirring for 3 h at 80 °C. FUR was left for 1 h in distilled water and then dissolved at 100 °C during stirring. After combining the solutions in appropriate ratios, the film-forming solution was poured onto Petri dishes and left under a fume hood to dry for 48 h.

To prepare CHIT–FUR=HGEL films, the CHIT–FUR complex was prepared in the same manner as the control, and afterwards, the mixture was stirred for 10 min at 50 °C and HGEL in concentrations of 0.25, 0.50 and 0.75% was added to obtain different coatings (HGEL 0.25, HGEL 0.50 and HGEL 0.75 respectively).

Glycerol in the concentration of 1% was added at the final stage of preparing the coating, followed by 10 min of stirring. Pouring and drying procedures were performed as described for the control.

#### 2.2.2. Film Characterisation

##### Fourier Transform Infrared Spectroscopy-Attenuated Total Reflection (FTIR-ATR)

The FTIR-ATR spectra of the newly-developed films were recorded within the range of 4000–700 cm^−1^ and 32 complete scans (resolution of 4 cm^−1^) were performed on the MATTSON 3000 FT-IR (Mattson Inc., Madison, WI, USA) spectrophotometer.

##### Differential Scanning Calorimetry (DSC)

Differential scanning calorimetry (DSC) analysis of the newly-developed films was performed using the DSC 204F1 differential scanning calorimeter (Phoenix Netsch, Selb, Germany). The samples were hermetically sealed in aluminium pans. Heating was performed at a rate of 10 °C/min within the temperature range of 25 to 300 °C. As a standard, an empty aluminium pan was used. The temperatures of enthalpy and thermal transition were calculated by means of computer software (Proteus Analysis, Netzsch GmbH, Selb, Germany) and enthalpy was expressed as J/g.

##### Surface Colour Measurement

Film colour was analysed using the CIE L * a * b * scale by means of the reflection method. The Colour i5 spectrometer (X-Rite, Grand Rapids, MI, USA) was configured for: d/8 measuring geometry, D65 illuminant, 10° observer and 25-mm measuring slot. Each sample was analysed using 5 replications. The total colour difference (Δ*E*) was calculated according to the following formula:

(1) $$\Delta E=\sqrt{{\left(\Delta L\right)}^{2}+{\left(\Delta a\right)}^{2}+{\left(\Delta b\right)}^{2}}$$

where Δ*E* is the colour total difference value of the CHIT–FUR film as a standard and those of the CHIT–FUR-HGEL.

##### Atomic Force Microscopy (AFM)

The Dimension Icon XR atomic force microscope (AFM) (Bruker, Santa Barbara, CA, USA) was used to obtain images. The microscope worked in the air via the Peak Force Tapping (PFT) mode. Standard silicon cantilevers with a 0.4 N/m constant nominal spring and <10-nm tip radius were applied. Prior to analysis, small pieces of each sample were glued to a smooth silicon wafer.

##### Antioxidant Activity

To measure antioxidant activity, aqueous film extracts at the concentration of 10 mg/mL were prepared. The extracts were heated up to 50 °C in a water bath and shaken for 10 min until complete dissolution of the two antioxidant assays: Ferric Reducing Antioxidant Power (FRAP) and 2,2-diphenyl-1-picryl-hydrazyl-hydrate (DPPH) were performed on the obtained film extracts. For FRAP analysis, the FRAP solution comprising an acetate buffer (pH 3.6), a 20-mM ferric chloride solution and 10-mM 2,4,6-tripyridyl-s-triazine solution (10 mM TPTZ in 40 mM HCl) at a ratio of 10:1:1 (*v*/*v*/*v*), was freshly prepared after each analysis. Before mixing with the film extract, the FRAP solution was incubated in the dark for 30 min at 37 °C. Afterwards, the FRAP extract was mixed with a film extract in the ratio of 0.4:3.6 (*v*/*v*) and the mixture was again incubated in the dark at 37 °C for 10 min and measured for its absorbance at 593 nm on the Helios Gamma UV-1601 spectrophotometer (Thermo Fisher Scientific, Waltham, MA, USA). For DPPH assay, the film extract was mixed with 0.1 mM DPPH in ethanol at the ratio of 0.2:2.8 (*v*/*v*), respectively. Following this, the mixture was incubated for 30 min in the dark, and then measured for its absorbance at 517 nm (Helios Gamma UV-1601, Thermo Fischer Scientific, Waltham, MA, USA). The results were compared with a blank sample (film extract substituted with distilled water). The results are expressed as % of inhibition and calculated using the following formula:

(2) $$DPPH\;radical\;scavenging\;\left(\%\right)=\frac{A_{blank}-A_{sample}\;}{A_{blank}}*100\%$$

where *A* is the absorbance of the *sample*/*blank* at 517 nm

The analyses were performed in 2 repetitions for 3 samples of the films (*n* = 2 × 3).

#### 2.2.3. Evaluation of Blueberry Storage Quality

##### Blueberry Treatments

To investigate the effectiveness of the developed active packaging, a special experiment was conducted using blueberries as a model food product, which were wrapped in each type of the investigated films. The control groups consisted of blueberries packed in LDPE film and unwrapped blueberries. Samples were stored at room temperature (~25 °C) for 10 days and periodically analysed for quality changes on days 3, 6 and 10 of storage.

##### Weight Loss Ratio

The weight loss was analysed according to the following formula:

weight loss (%) = [(W_0_ − W_t_) ÷ W_0_] × 100 (3)

where W_0_ is the initial weight of the blueberries; W_t_ is the weight of the blueberries on the day of measurement.

##### Determination of Total Phenolic Content (TPC)

The blueberry extract was prepared by adding 2.5 g of homogenised blueberries into 20 mL of a 70% ethanol solution. The mixtures were submerged in an ultrasonic bath (Polsonic, Palczyński, Warsaw, Poland) for 30 min, filtered, transferred in 50-mL volumetric flasks and filled up to 50 mL with the 70% ethanol solution. Determination of the total phenolic content was performed using the Folin–Ciocalteu method [22]. Moreover, 0.5 mL of the extract was mixed with 2.5 mL of the Folin–Ciocalteu reagent. After 3 min of incubation, 2 mL of the Na_2_CO_3_ (20%) solution were added, and the mixture was left for 2-h incubation in the dark. Afterwards, the samples were measured for their absorbance at a 760-nm wavelength (Helios Gamma UV-1601, Thermo Fisher Scientific, USA). The calibration curve was based on the gallic acid solutions, and the results are expressed as milligrams of gallic acid equivalents per litre of the extract (mg GAE/L).

##### Colour Determination of Blueberries

During each day of analysis, the 5 randomly selected blueberries were analysed for changes in their surface colour. The analysis was performed on the CR 200 Minolta Chroma Meter (Konica Minolta, Osaka, Japan), using the CIE Lab scale. The analysis of each blueberry consisted of 3 readings on 2 opposite sides of the equatorial region.

#### 2.2.4. Statistical Analysis

All analyses were performed using 3 independent repetitions with each analysis performed in duplicate, unless otherwise stated. Normality regarding distribution of the results was checked using the Shapiro–Wilk test. Box–Cox transformation was used in the case of variables with non-normal distribution. The results were subjected to one-way ANOVA (in the case of film determination) or two-way ANOVA (in the case of analyses on blueberries, with film type and storage time used as independent variables) with Tukey’s test to determine differences between the groups. The null hypothesis was discarded for *p* < 0.05.

## 3. Results and Discussion

### 3.1. Characterisation of CHIT, FUR Films and Their Complex with HGEL

**Figure 1:**
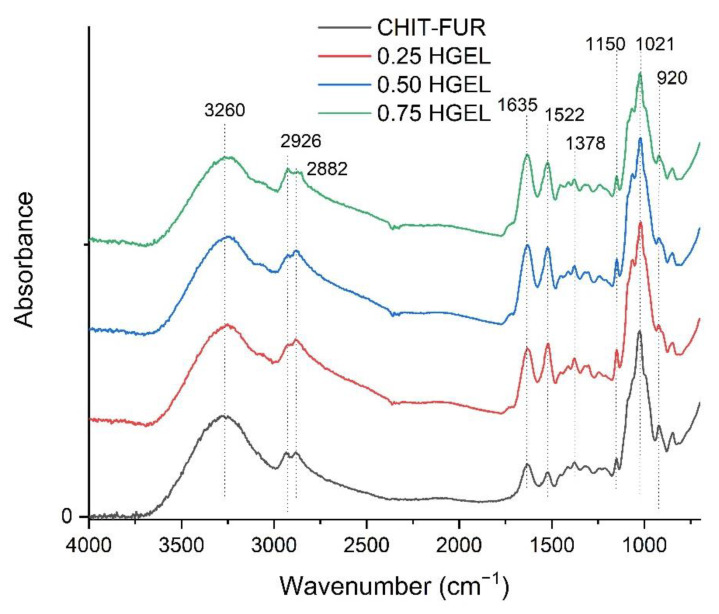
The FTIR-ATR spectrum of furcellaran, chitosan and their complexes with HGEL.

In Figure 1, the FTIR spectra of CHIT–FUR and CHIT–FUR/HGEL films are shown. Potential interactions between chitosan and furcellaran have been discussed in previous research [20]. The CHIT–FUR complex was prepared as a film matrix for the incorporation of the gelatin hydrolysate.

In multicomponent systems, such as the ones presented in this paper, many characteristic bands overlap and broaden. A wide band from approx. 3130 to 3500 cm^−1^ corresponds to –OH stretching absorption peaks of chitosan, furcellaran and amine NH symmetric vibration. In turn, the bands at 2926 and 2882 cm^−1^ correspond to CH stretching, while the peak at 1378 cm^−1^ is assigned to vibration of C-H. Within the range from 1150–920 cm^−1^, we can observe C-N stretching vibrations overlapping the vibrations from the carbohydrate ring. The addition of HGEL to the CHIT–FUR film caused an increase in the intensity of the peaks at ~1635 cm^−1^ (amide-I, CO and CN) and ~1522 cm^−1^ (amide-II). Such behaviour may indicate an interaction between the free negative sulphate groups of FUR and the positively charged amide groups of HGEL [19]. In addition, slight shifts and changes in peak intensity were observed, while no new peak was noted in the CHIT–FUR-HGEL composite layer compared to the pure CHIT–FUR one, indicating that the film components were compatible. Zhang, et al. [23] came to similar conclusions obtaining chitosan-based films and a rapeseed protein hydrolysate.

### 3.2. Thermal Properties of CHIT–FUR and CHIT–FUR/HGEL

The thermal properties of the films are important features in the selection of storage and processing conditions, as well as potential application. From this point of view, the thermal characteristics of polysaccharide-based films can provide a better understanding of the polysaccharide film distribution as well as information on the stability of biopolymer films [24]. The DSC parameters of the melting peak: temperature (T_m_) and the transition enthalpy change (∆H) of tested films are shown in Table 1. The melting temperature values ranged from 190.3 °C for the film based on the CHIT–FUR complex to 201.6 °C for the film with 0.5% HGEL addition. However, the performed statistical analysis showed that the mean values of melting points for the analysed films did not significantly differ. Nonetheless, it was found that values of melting enthalpy concerning films with the HGEL addition were significantly lower compared to the pure CHIT–FUR matrix, which shows that the presence of HGEL in the film structure reduces the amount of heat needed for structure melting of CHIT–FUR/HGEL complexes. The statistical differences between the average ∆H values for films with different HGEL levels were slightly differentiated. The ∆H parameter depends on the level of crystallinity because it increases with the degree of crystallisation. Moreover, the total rate of crystallisation at a given temperature decreases with increasing defects of the spatial structure [25]. Among composites with the addition of HGEL, the lowest value of the melting enthalpy was characterised by the film with the lowest 0.25% of its share, which indicates that the small presence of the added peptide to the greatest extent loosens the structure of the composite, lowering its order, and thus, decreasing its thermal stability. A further increase in HGEL addition caused a slight increase in ΔH value, however, no significant differences were found between composites with 0.25% and 0.5%, or 0.5% and 0.75% HGEL additions. Although the films with HGEL were characterised by lower thermal stability compared to the pure CHIT–FUR matrix, the increasing addition of HGEL improved this stability, which may be the result of mutual interactions between the polymers forming the composite structure.

### 3.3. Colour Properties of the Tested Films

The colour of the packaging is important for overall appearance and consumer acceptance [26]. The appearance of the packaging is one of the factors determining the purchase of food products [27].

For this purpose, the colour analysis of pure CHIT–FUR films and three variants with the addition of hydrolysate was conducted. On the basis of visual assessment, it was found that the binary film based on chitosan/furcellaran was transparent and slightly yellowish in colour, derived from the natural bile of these polysaccharides. The colour of the obtained films is consistent with the results of research on this type of materials published to date [8]. In the case of the matrix with the addition of HGEL, the films were slightly brown, which can be attributed to the natural colour of the hydrolysate. The changes in the L, a* and b* coordinates are given in Table 2, where it can be seen that the inclusion of HGEL in the CHIT–FUR film caused changes in the appearance of the tested films. The differences in brightness between the samples are small but statistically significant. Films with the addition of HGEL are slightly darker than the pure matrix and darker with increasing HGEL concentration. Such a colour change results from the colour of the gelatin hydrolysate, the colour of which is determined by inorganic, protein and other compounds introduced or not removed during its production [28]. The a* parameter had negative values, which means that the green colour occurs with different intensity for each variant. All the film variants showed positive values for the b* parameter. It was observed that the yellowness increased significantly with the addition of HGEL compared to the pure matrix, however, the data in Table 2 allow to indicate that the b* parameter is the same for the control and the CHIT–FUR/HGEL 0.75 films but differs from the HGEL 0.25 and HGEL 0.5 samples. Such a difference may be caused by several factors, mainly the natural colour of the gelatin hydrolysate and the uneven distribution of HGEL in the polysaccharide matrix. Moreover, such a colour change of the film may indicate too much gelatin hydrolysate for the furcellaran–chitosan complex. Increasing the amount of gelatin hydrolysate makes the films more opaque, which is related to the colour of the hydrolysate.

### 3.4. Atomic Force Microscopy (AFM)

**Figure 2:**
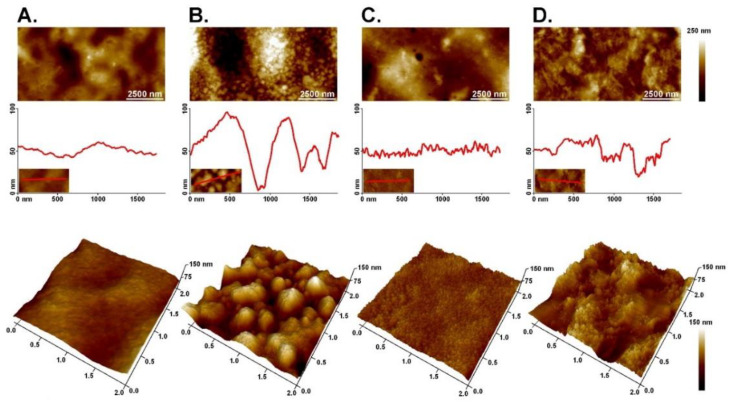
AFM topography images with corresponding cross-sections: (**A**) CHIT–FUR, (**B**) 0.25 HGEL, (**C**) 0.50 HGEL and (**D**) 0.75 HGEL.

In recent years, atomic force microscopy (AFM) methods have become comprehensive analytical tools for measuring surface characteristics and mechanics of both synthetic and biological materials. Using this method, high-resolution, three-dimensional surface imaging of materials and the characteristics of nano-scale topography can be obtained [29]. The topography of CHIT–FUR as well as CHIT–FUR films enriched with HGEL in various concentrations is shown in Figure 2.

Various pore shapes, including those round, elliptical and slit-shaped, were observed in AFM images of the membrane surface. In Figure 2A, the CHIT–FUR binary matrices are shown—the surface is smooth and even. In previous studies, it has been demonstrated that out of the three tested combinations, the 9:1 composite had the smoothest surface, which may indicate that at this ratio, the interaction of atoms between FUR and CHIT is optimal [20]. In Figure 2B–D, matrices enriched with HGEL in the proportions 0.25, 0.50 and 0.75 are shown. It is clearly visible that the addition of HGEL at each concentration caused irregular surface morphology, especially in the case of the 0.25 and 0.75 HGEL additions. Such differences may result from the selection of the appropriate amount of concentrations. Based on this study, it can be assumed that at the concentration of 0.25 HGEL is too low and becomes entirely combined with polysaccharides to form larger aggregates, while the addition of 0.5 optimally binds to the matrix without disturbing its structure. In the case of 0.75, imaging allows to suggest that HGEL binds similarly to 0.5, however, the matrix is oversaturated with the additive, which led to surface structure disturbance, but not as much as in the case of a too low concentration. AFM imaging clearly showed the presence of grooves and cracks on the surface of the film. All parameters indicated a clear increase in the degree of roughness for the B-D samples. The differences in the surface may result from the structure of the hydrolysate and its strong influence on the structure of the CHIT–FUR binary film.

### 3.5. Antioxidant Properties

The antioxidant properties and the ability to scavenge free radicals of CHIT–FUR and CHIT–FUR/HGEL films are presented in Table 3.

All samples with the addition of HGEL had a greater ability to deactivate the DPPH radical and reduce FRAP compared to the matrix, and with the increase of the concentration of HGEL, the antioxidant activity of the coatings increased. The addition of 0.25 HGEL to the CHIT–FUR matrix already caused a significant increase in the DPPH scavenging activity to a level above 53%. The results of FRAP analysis regarding the tested allowed to indicate a similar trend. The control CHIT–FUR matrix has only slight antioxidant properties, which are probably related to the antioxidant potential of chitosan [30]. On the other hand, a small addition of gelatin hydrolysate significantly improves the ability to both scavenge free radicals and reduce iron ions. The highest antioxidant activity measured via the FRAP method was characteristic for films containing the highest concentration of HGEL in the matrix. As the concentration of HGEL in the matrix increases, the antioxidant properties also increase. The antioxidant capacity of the gelatin hydrolysate results from the presence of peptides in it, which may have an antioxidant effect, mainly the Ala-Tyr peptide [17]. The addition of HGEL improved the scavenging activity of DPPH. In previous research, furcellaran films with the addition of gelatin hydrolysates from carp skins showed poor scavenging properties for DPPH free radicals. Therefore, it may be assumed that interactions took place in the furcellaran-hydrolysate matrix, which limit the antioxidant effect of the hydrolysate [31]. Such interactions between the CHIT–FUR matrix and the hydrolysate comprising gelatin from carp skins, presented in this research, were not observed, which proves the high compatibility of the film components.

### 3.6. Application on Model Food—Blueberry

**Figure 3:**
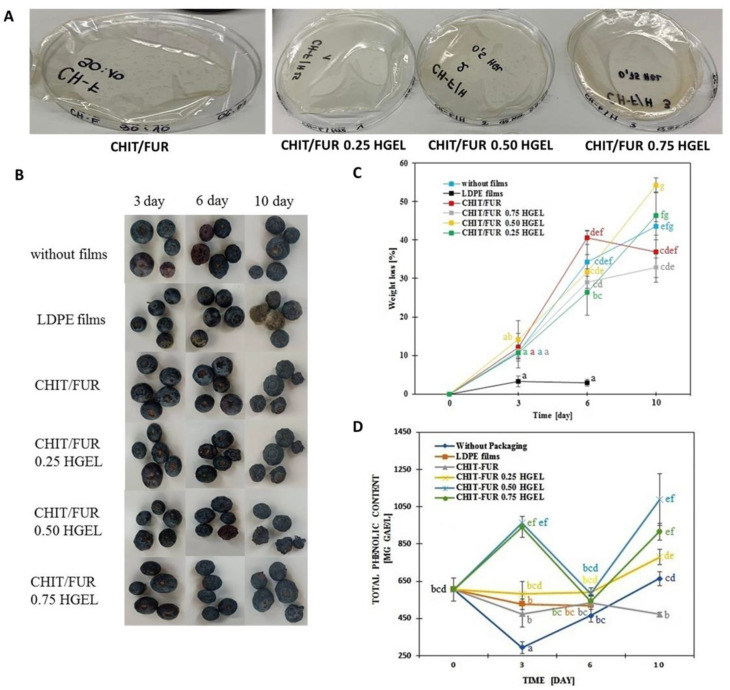
Visual appearance (**A**) of tested films, (**B**) of blueberries after storage, (**C**) weight loss and (**D**) total phenolic content of blueberry storage in different films. The same superscript letters in point demonstrate lack of significant difference between values (*p* ˂ 0.05).

On the basis of the results presented above, it was found that the analysed coatings have the potential to be used as active packaging materials. In further research, blueberries were used as a model food product, which are easily available and have a short shelf-life. In this study, the fruit was packed in three types of films: LDPE, CHIT–FUR and CHIT–FUR with the addition of HGEL in three concentrations. For comparative purposes, the American blueberry fruit not packed in any packaging material was also analysed (Figure 3).

In Figure 3A, the appearance of blueberry samples is demonstrated during 10 days of storage. There were no visual differences among the remaining coated groups. On the 10th day of storage, the fruits covered with synthetic film were completely mouldy, which made it impossible to assess their weight loss, polyphenol content or colour parameters. Lack of mould on the fruits not covered and in HGEL films may have been due to reduced water activity connected with weight loss via migration of water to its surrounds. It can therefore be assumed that the LDPE film retains water vapour better, limiting its migration, which contributed to the creation of conditions favourable for the growth of microorganisms [32].

Weight loss is an important index of post-harvest storage life in fresh fruits. This is primarily due to the loss of water during metabolic processes such as transpiration and respiration. Both processes are affected by the storage environment of the fruit, and the loss in weight is an indicator of how the product is handled and stored [33]. Blueberries covered with biodegradable coatings and fruit without packaging showed significantly higher weight loss during the entire storage period compared to fruit covered with synthetic film (Figure 3B). However, the fruit covered with synthetic film was the only one among the studied groups to become mouldy on the 10th day of storage. Fruit weight loss during storage is mainly due to water migration from the fruit into the environment [34]. Fruit spoilage is due to the accumulation of moisture in the unventilated film, which can promote rotting and the growth of mould on the surface of packaged products. The weight loss of blueberries packed in biodegradable films was similar to the weight loss of those without any coating. Among the films with the addition of HGEL, all samples showed progressive weight loss throughout the storage period. Nonetheless, the least weight loss of blueberries was observed when using films with HGEL 0.75 (32% during the entire storage period). The greatest weight loss, as much as 54%, was recorded on day 10 for the fruit packed in HGEL 0.5 films. Similar results were obtained by Duan, et al. [35] using edible coatings of chitosan, calcium caseinate and sodium alginate regarding the quality of fresh blueberries during storage. They observed an increase in weight loss for both blueberry varieties throughout the entire storage period in room temperature. The weight loss study showed that only by storing blueberries in HGEL 0.75, the weight loss was lower than in the case of fruit stored without coatings. Nonetheless, these differences between groups were not statistically significant.

One of the main problems associated with the storage of blueberries is their short shelf-life and the degradation of polyphenols during this period. The degradation of health-related compounds during storage can often be more severe than those observed during processing [36]. The total polyphenol content is shown in Figure 3C.

Packed blueberries contain 0.50 and 0.75% of protein hydrolysate and generally had a higher total phenolic content than those without coating or packed with synthetic or control films. This might be because the packed fruit ripened faster than that without treatment and the control group. Post-harvest storage has impact on phenolic content, while enzymes play a significant role in phenolic metabolism. During storage, the post-harvest ripening process continued faster in fruits packed with the novel films under study. According to data from literature, fruits may perceive coating materials as potential abiotic stress, thus resulting in the production of secondary metabolites such as phenols in packed samples [33]. In previous studies, it has been revealed that a chitosan coating could decrease the loss of phenolic compounds and the occurrence of browning in fruit [37]. In the study by Yang, et al. [38], the chitosan coating with blueberry leaf extract helped retain the total phenolic content in blueberries. On the basis of the obtained results, it can be concluded that the fruit packed in coatings with 0.50 and 0.75% addition of the gelatin hydrolysate from carp skin ripen faster compared to that stored in the chitosan-furcellaran film, which results in an increase in their polyphenol content. According to Alvarez, et al. [39], the presence of antioxidants could have a protective effect against oxidation and, at the same time, contribute to the activation of producing phenolic compounds by the fruit tissues, similarly as the coatings with the addition of the carp skin gelatin hydrolysate characterised by high antioxidant properties analysed in this work.

One of the most important parameters to which consumers are sensitive when selecting foods is their colour [36]. In Table 4, there is a list regarding the colour attributes of blueberries in different coatings during a 10-day storage period.

No differences were observed between a* values of blueberries after 10 days of storage. Statistically significant changes in L were noted for fruit without the packaging on day 6 compared to fruit in the HGEL 0.25 film on day 10. In case of the b* parameter, HGEL 0.75 on day 6 was significantly lower than in fruits without packaging, packed in LDPE and HGEL 0.25 on day 3 and packed in HGEL 0.25 and 0.75 on day 9. Due to lack of a clear trend, those differences could have been related more to sample diversity than to the effect of coating. It has been reported that the colour parameter can be affected by the natural waxy layer covering the berry surface, known as bloom [40]. Blueberry bloom contains various lipidic components, mainly triterpenoids and diketones. Their main function is to protect the fruit against external agents, to prevent its dehydration and softening, etc. [40]. According to Abugoch, et al. [41], colour in fresh blueberries is determined by the waxy bloom rather than by the pigment content. The active coatings that were tested did not contain free lipophilic compounds that could react with the components of the waxy bloom berries, and thus, they did not affect the colour parameter. Yang, Yue, Gong, Qian, Wang, Deng and Zhao [38] also found no effect of active chitosan coatings on changes in the blueberry colour. Similarly in the research by Bambace, et al. [42], the alginate-based coatings enriched with inulin and oligofructose did not affect the colour parameters of the blueberries covered with them.

## 4. Conclusions

We successfully created a three-component complex consisting of two polysaccharides and one protein component. The films based on furcellaran (FUR) and chitosan (CHIT) enriched with the gelatin hydrolysate (HGEL) were obtained, without changes in the structural level of the CHIT–FUR film, which was confirmed by FT-IR testing. On the other hand, in relation to the control film, AFM imaging showed the presence of grooves and cracks on the surface of the film to which the hydrolysate had been introduced. The hydrolysate deteriorated the thermal stability of the film and gave the matrix antioxidant properties, which was confirmed by FRAP and DPPH tests. Due to their properties, the resulting films demonstrated potential as active packaging materials for food storage. Blueberry fruit packed in the tested coatings did not become mouldy during the 10 days of storage, unlike the fruit covered with synthetic film. In contrast, the films did not inhibit the reduction in fruit weight. This may indicate lower gas permeability of the CHIT–FUR/HGEL film compared to the synthetic one, which allowed less water vapour to pass compared to the biopolymer film further causing the fruit to become mouldy. Nonetheless, it did not lose its weight. Based on the results of the polyphenol content, it can be assumed that fruit packed in coatings with the 0.50% and 0.75% addition of gelatin hydrolysate from carp skins ripen faster compared to fruit stored in chitosan-furcellaran film. The obtained results indicate the developmental nature of the work—it is necessary to investigate the influence of active packaging materials on the storage of other types of food products. In addition, a separate sensory test of the stored fruit should be carried out in the future to assess whether these edible coatings influence the most important quality characteristics such as taste, aroma, texture and appearance.

## Figures and Tables

**Table 1:** Thermal properties of films CHIT–FUR and with addition of the HGEL.

Film type	Peak Temperature (T_m_) (°C)	Enthalpy (ΔH_m_) (J/g)
CHIT–FUR	190.3 ^a^ ± 8.6	154.80 ^c^ ± 4.2
HGEL 0.25	198.0 ^a^ ± 11.6	122.57 ^a^ ± 2.5
HGEL 0.50	202.4 ^a^ ± 5.7	129.23 ^ab^ ± 3.9
HGEL 0.75	201.6 ^a^ ± 4.5	138.57 ^b^ ± 4.5

The same superscript letters in each column demonstrate lack of significant difference between values (*p* > 0.05). Values are expressed as mean ± SD.

**Table 2:** Colour properties of CHIT–FUR films and their blends with HGEL.

Film Type	L	a*	b*	ΔE
9:1 CHIT–FUR	91.99 ^d^ ± 0.38	−1.54 ^b^ ± 0.02	18.81 ^a^ ± 0.36	-
HGEL 0.25	89.26 ^c^ ± 0.51	−1.64 ^b^ ± 0.17	30.07 ^b^ ± 1.41	11.59
HGEL 0.50	85.63 ^b^ ± 1.0	−0.93 ^a^ ± 0.18	32.20 ^b^ ± 1.41	15.04
HGEL 0.75	90.65 ^a^ ± 0.70	−0.94 ^a^ ± 0.04	18.17 ^a^ ± 0.86	1.74

The same superscript letters in each column demonstrate lack of significant difference between values (*p* > 0.05). Values are expressed as mean ± SD.

**Table 3:** Antioxidant activity of CHIT–FUR and CHIT–FUR/HGEL films with RE in different concentrations.

Film Type	DPPH (%)	FRAP (μmol Trolox/g of Dried Film)
CHIT–FUR	2.53 ^a^ ± 0.21	0.34 ^a^ ± 0.11
HGEL 0.25	53.63 ^b^ ± 0.44	1.11 ^b^ ± 0.31
HGEL 0.50	61.17 ^c^ ± 0.99	1.19 ^b^ ± 0.25
HGEL 0.75	60.32 ^c^ ± 0.02	1.85 ^c^ ± 0.15

The same superscript letters in each column demonstrate lack of significant difference between values (*p* > 0.05). Values are expressed as mean ± SD.

**Table 4:** The colour parameter of blueberries stored in different films.

		Without Packaging	LDPE Films	CHIT–FUR	HGEL 0.25	HGEL 0.50	HGEL 0.75
Day 0	L	32.65 ^ab^ ± 1.78					
	a*	−0.20 ± 0.14					
	b*	−5.23 ^ab^ ± 0.79					
Day 3	L	32.48 ^ab^ ± 1.81	32.59 ^ab^ ± 1.32	32.01 ^ab^ ± 2.49	31.02 ^ab^ ± 1.74	32.04 ^ab^ ± 2.27	29.63 ^ab^ ± 2.45
	a*	1.48 ± 0.95	−0.09 ± 0.13	0.10 ± 0.44	1.01 ± 0.65	0.44 ± 0.19	0.48 ± 0.48
	b*	−3.43 ^b^ ± 1.42	−3.85 ^b^ ± 2.69	−4.85 ^ab^ ± 1.48	−3.51 ^b^ ± 0.70	−4.21 ^ab^ ± 0.93	−2.95 ^b^ ± 1.22
Day 6	L	34.12 ^b^ ± 1.35	31.04 ^ab^ ± 2.12	30.74 ^ab^ ± 2.03	30.22 ^ab^ ± 1.94	30.79 ^ab^ ± 2.65	32.62 ^ab^ ± 1.06
	a*	1.94 ± 1.65	0.07 ± 0.63	0.01 ± 0.24	0.65 ± 0.60	1.17 ± 1.55	0.01 ± 0.33
	b*	−5.62 ^ab^ ± 0.91	−5.32 ^ab^ ± 1.30	−5.81 ^ab^ ± 0.59	−5.70 ^ab^ ± 0.54	−5.00 ^ab^ ± 1.20	−7.15 ^a^ ± 0.88
Day 10	L	32.76 ^ab^ ± 1.58		29.85 ^ab^ ± 1.23	28.06 ^a^ ± 3.14	29.06 ^ab^ ± 1.68	32.61 ^ab^ ± 1.75
	a*	0.25 ± 0.53		−0.27 ± 0.04	0.12 ± 0.28	0.68 ± 1.06	0.06 ± 0.16
	b*	−4.50 ^ab^ ± 1.37		−4.67 ^ab^ ± 0.55	−3.31 ^b^ ± 1.39	−3.48 ^b^ ± 1.45	−4.50 ^ab^ ± 0.49

The same superscript letters in each column demonstrate lack of significant differences between values (*p* > 0.05). Values are expressed as mean ± SD.

## Data Availability

Not applicable.

## References

[1] Ivonkovic A., Zeljko K., Talic S., Lasic M. (2017). Biodegradable packaging in the food industry. J. Food Saf. Food Qual.

[2] Zhong Y., Godwin P., Jin Y., Xiao H. (2019). Biodegradable polymers and green-based antimicrobial packaging materials: A mini-review. Adv. Ind. Eng. Polym. Res..

[3] Luangapai F., Peanparkdee M., Iwamoto S. (2019). Biopolymer films for food industries: Properties, applications, and future aspects based on chitosan. Rev. Agric. Sci..

[4] Cazón P., Velazquez G., Ramírez J.A., Vázquez M. (2016). Polysaccharide-based films and coatings for food packaging: A review. Food Hydrocoll..

[5] Garavand F., Rouhi M., Razavi S.H., Cacciotti I., Mohammadi R. (2017). Improving the integrity of natural biopolymer films used in food packaging by crosslinking approach: A review. Int. J. Biol. Macromol..

[6] Dharmalingam K., Anandalakshmi R., Katiyar V., Gupta R., Ghosh T. (2019). Polysaccharide-Based Films for Food Packaging Applications. Advances in Sustainable Polymers: Processing and Applications.

[7] Tang X.Z., Kumar P., Alavi S., Sandeep K.P. (2012). Recent Advances in Biopolymers and Biopolymer-Based Nanocomposites for Food Packaging Materials. Crit. Rev. Food Sci. Nutr..

[8] Júnior L.M., Vieira R.P., Jamróz E., Anjos C.A.R. (2021). Furcellaran: An innovative biopolymer in the production of films and coatings. Carbohydr. Polym..

[9] Imeson A.P., Phillips G.O., Williams P.A. (2009). 7—Carrageenan and furcellaran. Handbook of Hydrocolloids.

[10] Jamróz E., Juszczak L., Kucharek M. (2018). Development of starch-furcellaran-gelatin films containing tea tree essential oil. J. Appl. Polym. Sci..

[11] Hosseinnejad M., Jafari S.M. (2016). Evaluation of different factors affecting antimicrobial properties of chitosan. Int. J. Biol. Macromol..

[12] Ahmad S.I., Ahmad R., Khan M.S., Kant R., Shahid S., Gautam L., Hasan G.M., Hassan I. (2020). Chitin and its derivatives: Structural properties and biomedical applications. Int. J. Biol. Macromol..

[13] Wang H., Qian J., Ding F. (2018). Emerging Chitosan-Based Films for Food Packaging Applications. J. Agric. Food Chem..

[14] Feng X., Wang X., Xing W., Yu B., Song L., Hu Y. (2013). Simultaneous Reduction and Surface Functionalization of Graphene Oxide by Chitosan and Their Synergistic Reinforcing Effects in PVA Films. Ind. Eng. Chem. Res..

[15] Youssef A.M., Abou-Yousef H., El-Sayed S., Kamel S. (2015). Mechanical and antibacterial properties of novel high performance chitosan/nanocomposite films. Int. J. Biol. Macromol..

[16] Jiang Y., Yin H., Zhou X., Wang D., Zhong Y., Xia Q., Deng Y., Zhao Y. (2021). Antimicrobial, antioxidant and physical properties of chitosan film containing *Akebia trifoliata* (Thunb.) Koidz. peel extract/montmorillonite and its application. Food Chem..

[17] Tkaczewska J., Bukowski M., Mak P. (2018). Identification of Antioxidant Peptides in Enzymatic Hydrolysates of Carp (*Cyprinus Carpio*) Skin Gelatin. Molecules.

[18] Tkaczewska J., Morawska M., Kulawik P., Zając M. (2018). Characterization of carp (*Cyprinus carpio*) skin gelatin extracted using different pretreatments method. Food Hydrocoll..

[19] Jancikova S., Jamróz E., Kulawik P., Tkaczewska J., Dordevic D. (2019). Furcellaran/gelatin hydrolysate/rosemary extract composite films as active and intelligent packaging materials. Int. J. Biol. Macromol..

[20] Jamróz E., Janik M., Juszczak L., Kruk T., Kulawik P., Szuwarzyński M., Kawecka A., Khachatryan K. (2021). Composite biopolymer films based on a polyelectrolyte complex of furcellaran and chitosan. Carbohydr. Polym..

[21] Tkaczewska J., Jamróz E., Kulawik P., Morawska M., Szczurowska K. (2019). Evaluation of the potential use of a carp (*Cyprinus carpio*) skin gelatine hydrolysate as an antioxidant component. Food Funct..

[22] Katırcı N., Işık N., Güpür Ç., Guler H.O., Gursoy O., Yilmaz Y. (2018). Differences in antioxidant activity, total phenolic and flavonoid contents of commercial and homemade tomato pastes. J. Saudi Soc. Agric. Sci..

[23] Zhang C., Wang Z., Li Y., Yang Y., Ju X., He R. (2018). The preparation and physiochemical characterization of rapeseed protein hydrolysate-chitosan composite films. Food Chem..

[24] Jamaludin J., Adam F., Rasid R.A., Hassan Z. (2017). Thermal studies on Arabic gum—Carrageenan polysaccharides film. Chem. Eng. Res. Bull..

[25] Burfield D.R., Loi P.S.T., Doi Y., Mejzík J. (1990). DSC studies of tactic polypropylenes: The correlation of polymer stereochemistry with thermal properties. J. Appl. Polym. Sci..

[26] Pereda M., Dufresne A., Aranguren M.I., Marcovich N.E. (2013). Polyelectrolyte films based on chitosan/olive oil and reinforced with cellulose nanocrystals. Carbohydr. Polym..

[27] Liu X., Xu Y., Zhan X., Xie W., Yang X., Cui S.W., Xia W. (2019). Development and properties of new kojic acid and chitosan composite biodegradable films for active packaging materials. Int. J. Biol. Macromol..

[28] Avena-Bustillos R., Olsen C., Olson D., Chiou B., Yee E., Bechtel P., McHugh T. (2006). Water Vapor Permeability of Mammalian and Fish Gelatin Films. J. Food Sci..

[29] Marinello F., La Storia A., Mauriello G., Passeri D. (2018). Atomic Force microscopy techniques to investigate activated food packaging materials. Trends Food Sci. Technol..

[30] El-Hack M.E.A., El-Saadony M.T., Shafi M.E., Zabermawi N.M., Arif M., Batiha G.E., Khafaga A.F., El-Hakim Y.M.A., Al-Sagheer A.A. (2020). Antimicrobial and antioxidant properties of chitosan and its derivatives and their applications: A review. Int. J. Biol. Macromol..

[31] Jamróz E., Kulawik P., Tkaczewska J., Guzik P., Zając M., Juszczak L., Krzyściak P., Turek K. (2020). The effects of active double-layered furcellaran/gelatin hydrolysate film system with Ala-Tyr peptide on fresh Atlantic mackerel stored at −18 °C. Food Chem..

[32] Rabea E.I., Badawy M.E.-T., Stevens C.V., Smagghe G., Steurbaut W. (2003). Chitosan as antimicrobial agent: Applications and mode of action. Biomacromolecules.

[33] Zekrehiwot A., Yetenayet B.T., Ali M., Abebe Z., Tola Y.B., Mohammed A. (2017). Effects of edible coating materials and stages of maturity at harvest on storage life and quality of tomato (Lycopersicon Esculentum Mill.) fruits. Afr. J. Agric. Res..

[34] Yaman O., Bayoιndιrlι L. (2002). Effects of an Edible Coating and Cold Storage on Shelf-life and Quality of Cherries. LWT Food Sci. Technol..

[35] Duan J., Wu R., Strik B.C., Zhao Y. (2011). Effect of edible coatings on the quality of fresh blueberries (Duke and Elliott) under commercial storage conditions. Postharvest Biol. Technol..

[36] Lafarga T., Aguiló-Aguayo I., Bobo G., Chung A.V., Tiwari B.K. (2018). Effect of storage on total phenolics, antioxidant capacity, and physicochemical properties of blueberry (*Vaccinium corymbosum* L.) jam. J. Food Process. Preserv..

[37] Campaniello D., Bevilacqua A., Sinigaglia M., Corbo M.R. (2008). Chitosan: Antimicrobial activity and potential applications for preserving minimally processed strawberries. Food Microbiol..

[38] Yang G., Yue J., Gong X., Qian B., Wang H., Deng Y., Zhao Y. (2014). Blueberry leaf extracts incorporated chitosan coatings for preserving postharvest quality of fresh blueberries. Postharvest Biol. Technol..

[39] Alvarez M.V., Ponce A.G., Moreira M.R. (2017). Influence of polysaccharide-based edible coatings as carriers of prebiotic fibers on quality attributes of ready-to-eat fresh blueberries. J. Sci. Food Agric..

[40] Bof M., Laurent F., Massolo F., Locaso D., Versino F., García M. (2021). Bio-Packaging Material Impact on Blueberries Quality Attributes under Transport and Marketing Conditions. Polymers.

[41] Abugoch L., Tapia C., Plasencia D., Pastor A., Castro-Mandujano O., López L., Escalona V.H. (2016). Shelf-life of fresh blueberries coated with quinoa protein/chitosan/sunflower oil edible film. J. Sci. Food Agric..

[42] Bambace M.F., Alvarez M.V., Moreira M.D.R. (2019). Novel functional blueberries: Fructo-oligosaccharides and probiotic lactobacilli incorporated into alginate edible coatings. Food Res. Int..

